# Supplementary figures and images for: Dimensional reduction of phenotypes from 53 000 mouse models reveals a diverse landscape of gene function
Source: Bioinform Adv. 2021 Oct 11;1(1):vbab026. doi: 10.1093/bioadv/vbab026 (PMC8633315; doi:10.1093/bioadv/vbab026)

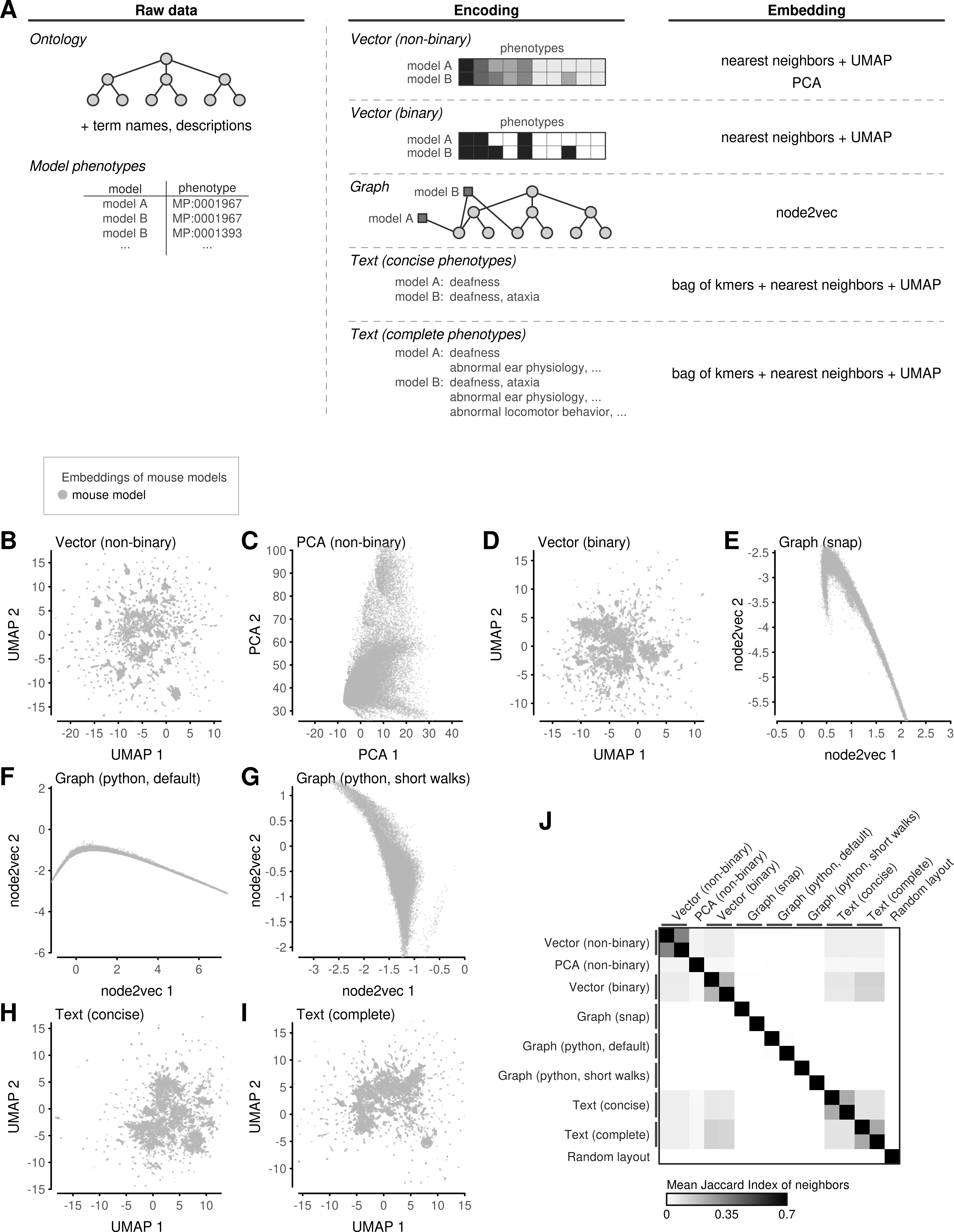

Supplement: vbab026_Supplementary_Data [file vbab026_supplementary_data.zip › FigS2.png]

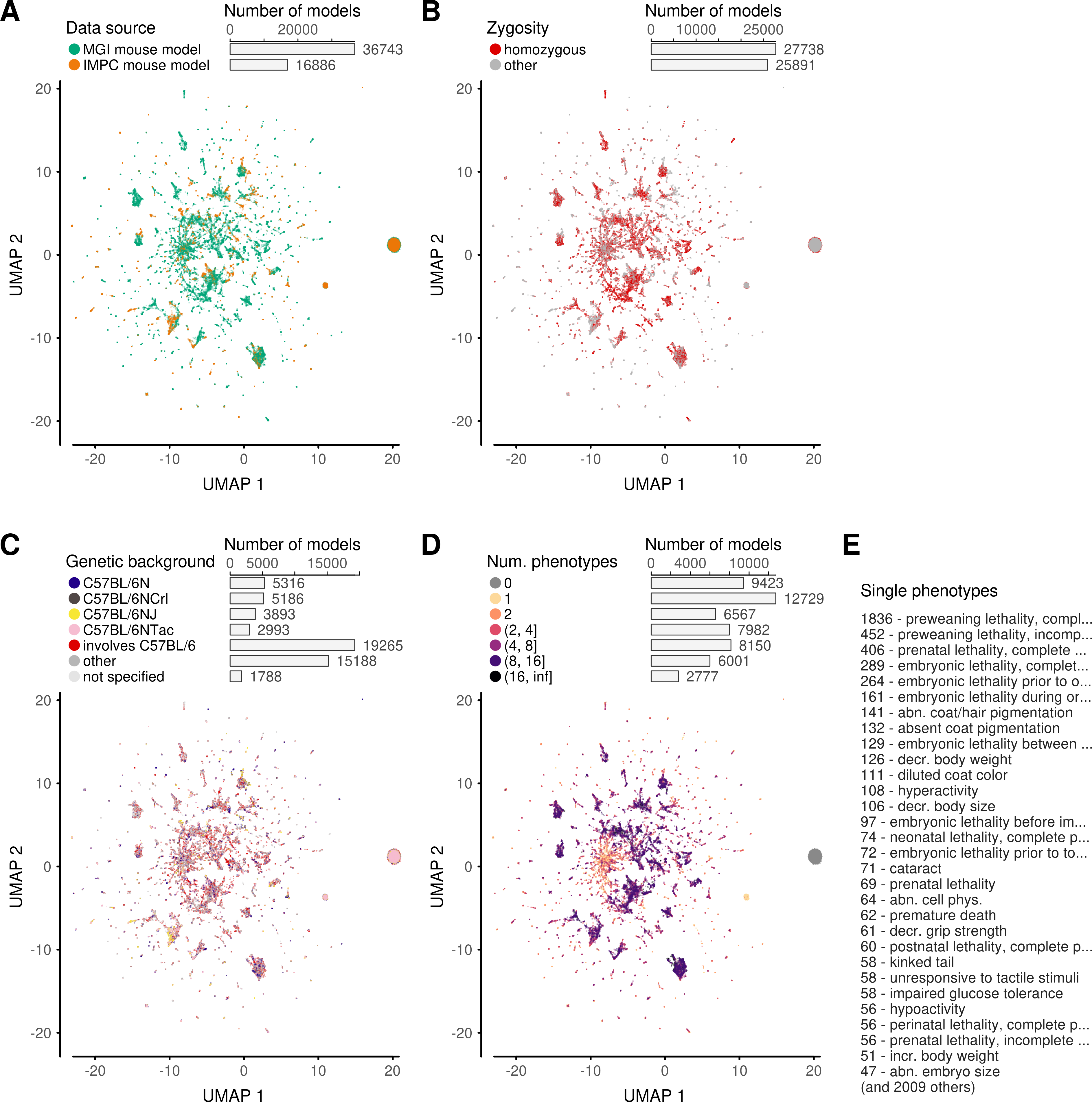

Supplement: vbab026_Supplementary_Data [file vbab026_supplementary_data.zip › FigS3.png]

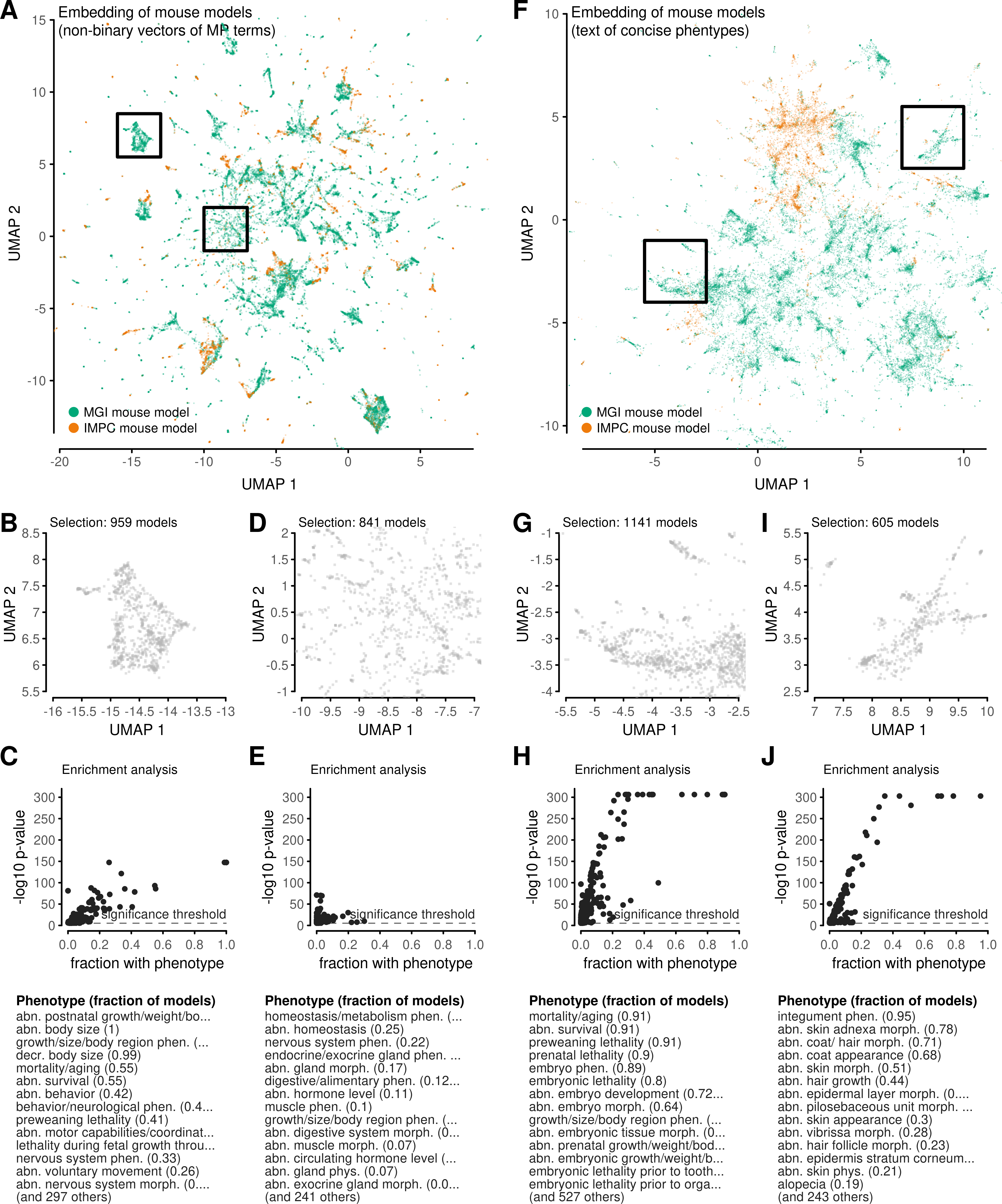

Supplement: vbab026_Supplementary_Data [file vbab026_supplementary_data.zip › FigS4.png]

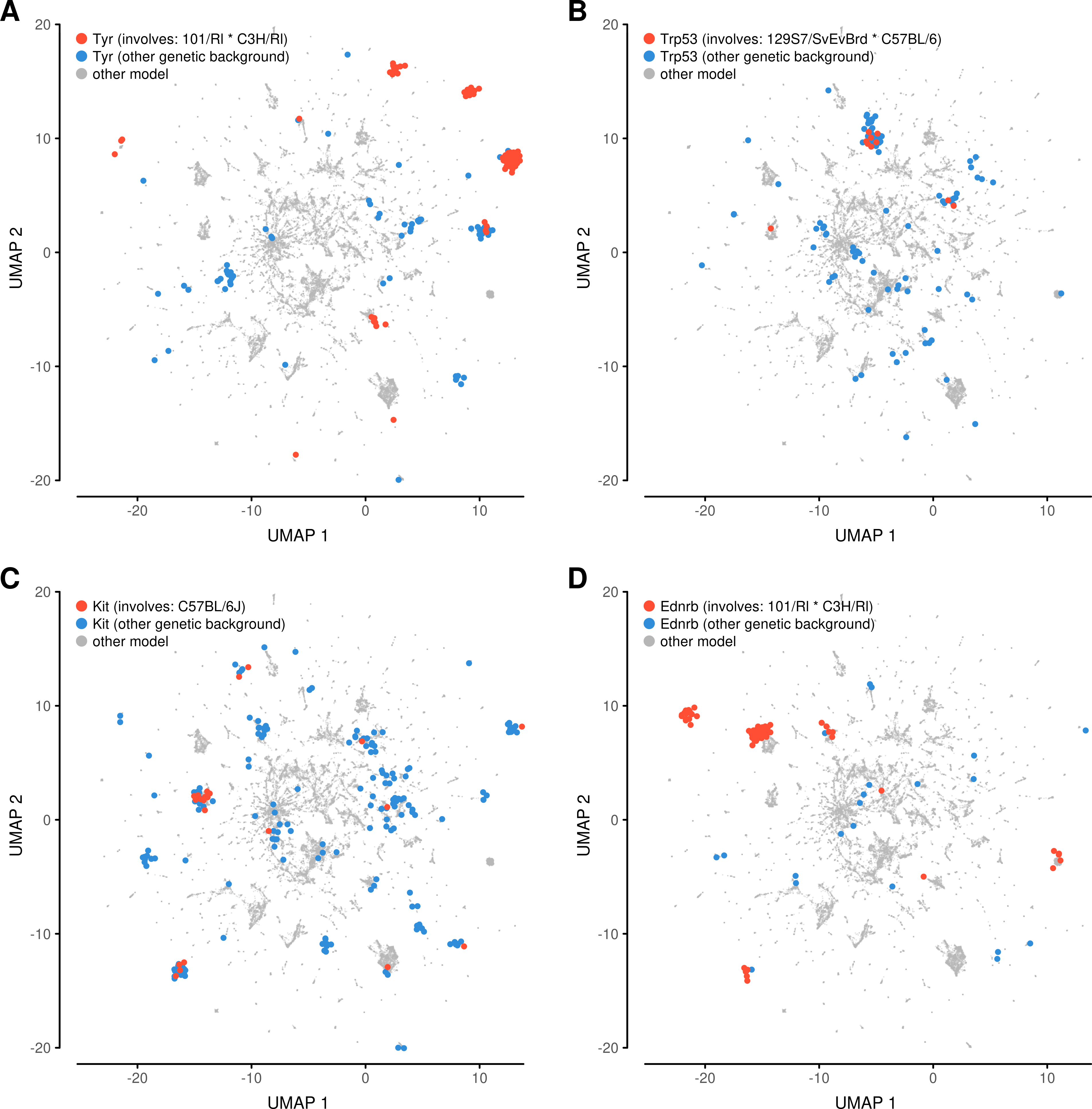

Supplement: vbab026_Supplementary_Data [file vbab026_supplementary_data.zip › FigS5.png]

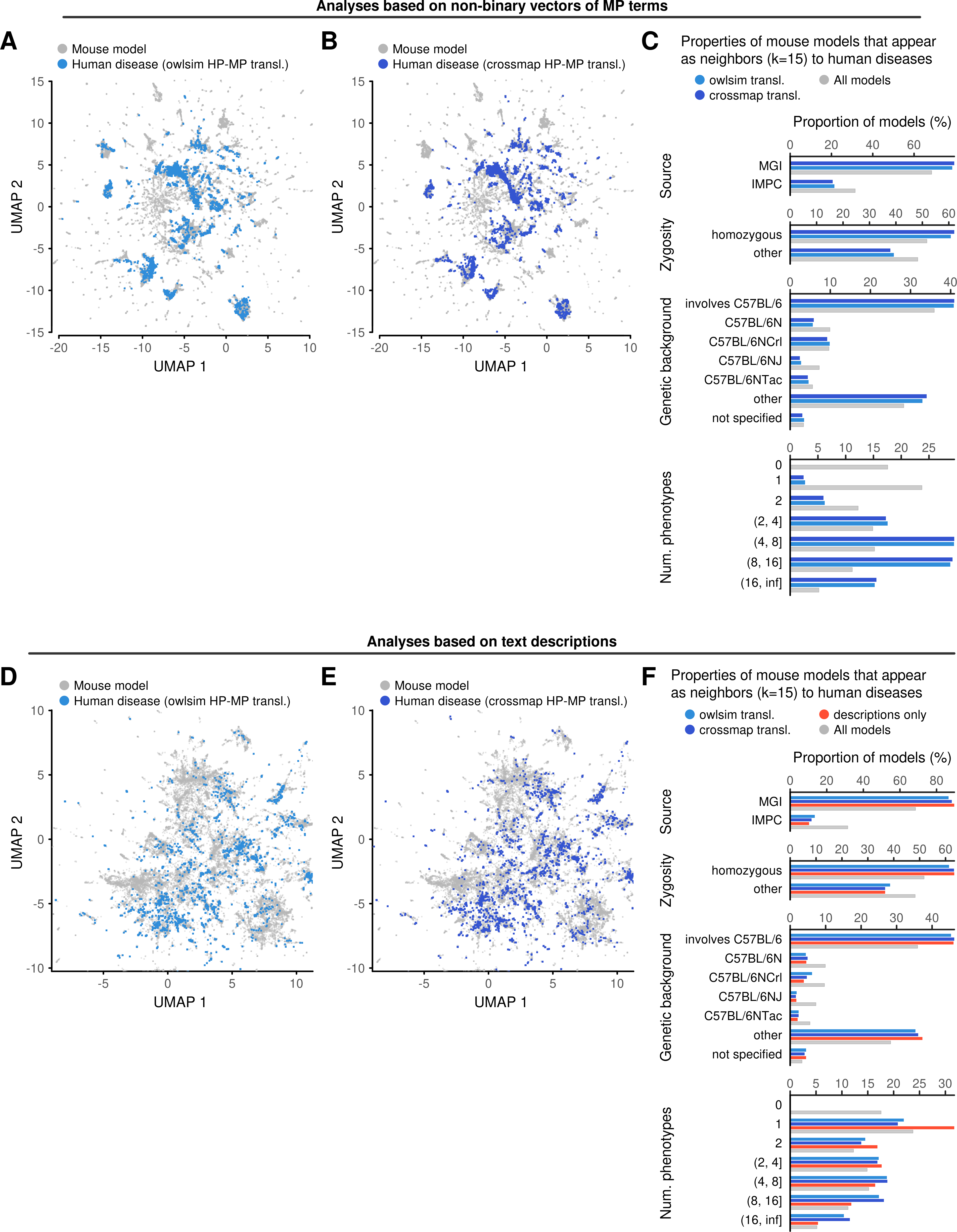

Supplement: vbab026_Supplementary_Data [file vbab026_supplementary_data.zip › FigS6.png]

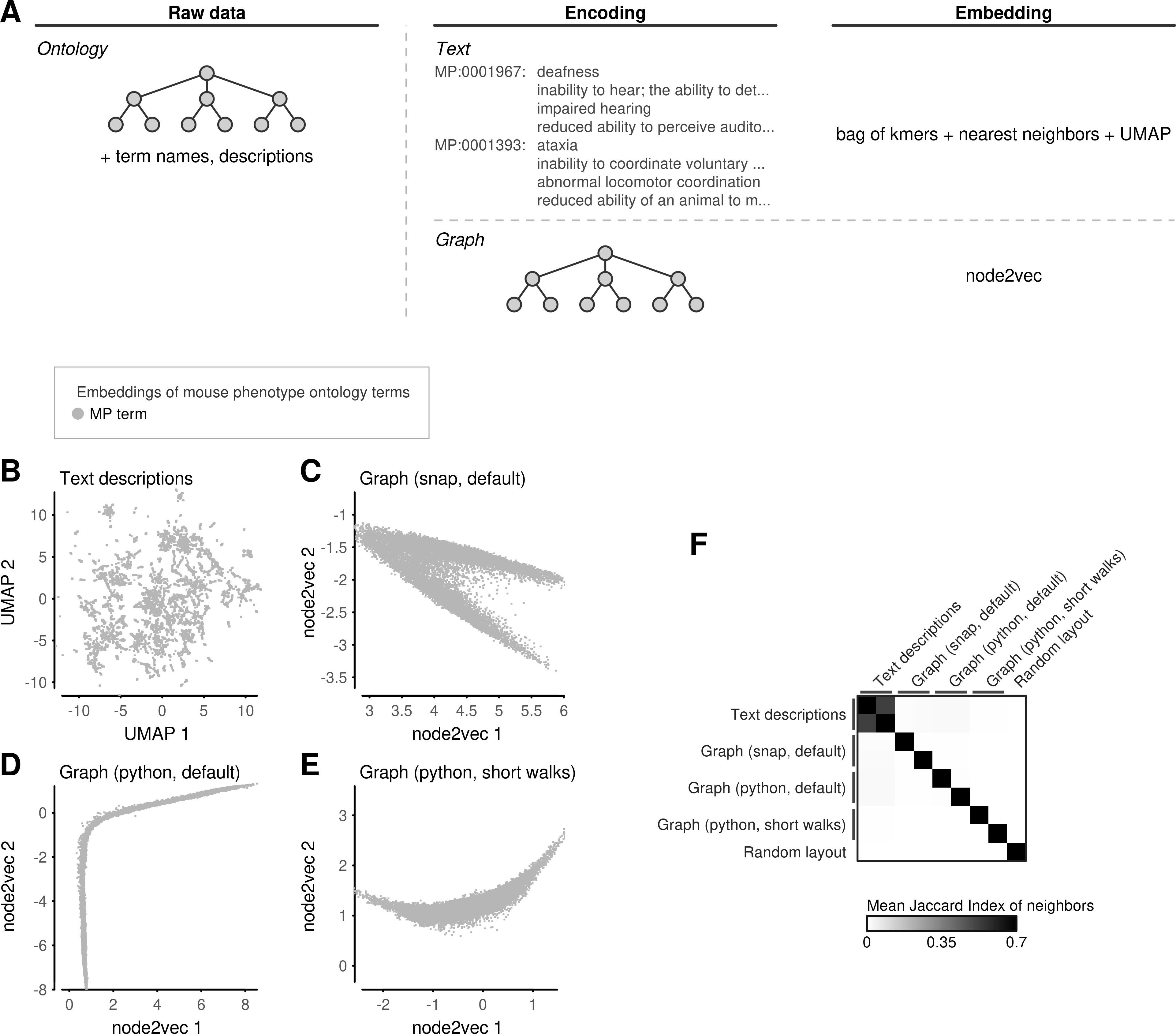

Supplement: vbab026_Supplementary_Data [file vbab026_supplementary_data.zip › FigS1.png]
